# Comparison of five different obturation approaches for resistance against fracture in root canal treated teeth

**DOI:** 10.6026/973206300200625

**Published:** 2024-06-30

**Authors:** Katya Pandey, Sulaima Mehar, Nitu Chaubey, Ajeet Kumar, Rajan Kumar Jha, Sagar Hiwale

**Affiliations:** 1 Department of Conservative Dentistry & Endodontics, Sri Aurobindo College of Dentistry, Indore, M.P., India; 2 Department of Conservative Dentistry & Endodontics, Mithila Minority Dental College and Hospital, Darbhanga, Bihar, India; 3 Department of Conservative Dentistry and Endodontics, Purvanchal Institute of Dental Sciences, GIDA, Gorakhpur, Uttar Pradesh, India; 4 Department of Conservative Dentistry & Endodontics, Inderprastha Dental College & Hospital, Ghaziabad, Uttar Pradesh, India

**Keywords:** Fracture resistance, obturation techniques

## Abstract

Endodontist should know about the fracture resistance of endodontic treated teeth in different obturation techniques to make a proper choice for obturation of mechano-chemically prepared root canals. Therefore, it is of interest to compare the fracture resistance of endodontically obturated teeth in five different obturation techniques namely single cone obturation (SCO), cold lateral compaction (CLC), cold free flow condensation, warm vertical compaction, injection molded thermo-plasticized technique. Among experimental categories, maximum fracture resistance was observed in cold free flow condensation technique while minimum fracture resistance was observed in injection molded thermo-plasticized technique. Cold free flow condensation technique using Gutta Flow2 has the potential to be used as obturation technique with minimum fracture resistance. Conventional techniques like CLC and SOC also offered good fracture resistance.

## Background:

In order to treat the root canal contamination and avoid or completely eradicate periapical spread of infection, root canal treatment is carried out [1, 2-3]. In order to avoid unsuccessful treatment caused by the reintroduction of bacteria and their byproducts into the canal system of the tooth and their discharge into the periapical tissue, three-dimensional closure of bio-mechanically prepared root canals is an essential first step [4, 5-6]. Since teeth that have been endodontically treated undergo dehydration and dentin erosion during mechano-chemical preparation, extended pharmaceutical exposure during process of disinfection and elevated pressure throughout obturation, these teeth are weaker and more prone to fracture than healthy teeth [7, 8-9]. Therefore, by adhering to and mechanically interconnecting root canal filling components with dentin of root, obturation of root canal aims to strengthen the root canal as well as enhance resilience to fractures of roots[10, 11-12].There are several obturation methods that can be used, such as single-cone obturation (SCO), thermoplastic gutta-percha, cold lateral compaction (CLC) and vertical condensation [13, 14-15].The CLC method has demonstrated positive clinical outcomes, is very safe, and is reasonably priced. It is the typical method for obturating root canals [16, 17, 18- 19]. Its disadvantages include a high degree of complexity, a chance of void development, and an increased likelihood of vertical fracture of root since wedging forces are applied using tools like spreaders [20, 21-22]. Insufficient obturation of twisted canals can also lead to a less-than-ideal result when using the CLC approach. The correct application of a spreader in the CLC technique may aid in creating additional room for the placement of auxiliary gutta-percha points [20, 21, 22, 23, 24- 25].Single cone obturation (SCO), which is a variant of the CLC approach [11- 16]. This method is well-liked since it is quick and easy to use, and it doesn't require compaction. Since this approach uses increasingly sealer compared to the compaction as well as condensation processes, its results are more dependent on the sealer's characteristics [12-17]. Gutta flow was first released in 2004. It is a newly developed flowable, nonheated GP system with the benefits of thermoplasticized GP systems, like homogenous bulk and less strains on roots, combined with the qualities of GP as well as sealer [16- 21]. Coltene unveiled the GuttaFlow2 root canal obturating system in 2012.GuttaFlow2, an improved version of the current GuttaFlow material, maintains the same superior material qualities. It is based on cold free flow compaction technique [12-19].Novel thermoplasticized injection equipment called the BeeFill system was created to make obturation easier following canal preparation using the Mtwo rotary system [5-11]. A heated vertical compaction system corresponds to the BeeFill system [7-14]. Endodontist should know about the fracture resistance of endodontic treated teeth in different obturation techniques to make a proper choice for obturation of mechano-chemically prepared root canals [12-19]. Therefore, it is of interest to compare the fracture resistance of endodontically obturated teeth in five different obturation techniques mentioned above.

## Methods and materials:

This in vitro study included two hundred and ninety teeth that were extracted within 48 hours. The teeth included in study were having single root with single canal and were mainly from anterior region of jaws (central incisors). The teeth selected for study had to accomplish following criteria

[1] Comparable root diameters

[2] Comparable root curvature

[3] Devoid of dental caries

[4] Without any fractures

[5] No restoration

[6] Not broken

[7] Without any displacements,

[8] Not subject to root resorption,

[9] No free open apices

After removing all debris and leftover tissues, the teeth were cleaned with a five percent solution of sodium hypochlorite and preserved in regular saline solution. Applying a diamond disc, crown of teeth was removed at the cementoenamel junction (CEJ) to provide a uniform root length of 14 mm. Size #15 K-files were utilized to determine the root length. Using digital radiography, the effective working length was adjusted to be 1.0 mm less than the real length of root canal. With the exception of the teeth specimen in the control category, all teeth were configured with M two rotary files (VDW) in a step-back manner up to size #25/0.06.

To get rid of the smear layer, root canals were treated with 10 milliliters of five percent NaOCl and subsequently 3 milliliters of seventeen percent Ethylenediaminetetraacetic Acid(EDTA). After that, a last flush was performed using 5 ml of regular saline and 1 ml of five percent NaOCl. Constant irrigation was used during the biomechanical preparation process. After that, samples were dried with #25/0.06 sterile paper points. The specimens were divided into different categories in following manner (Table 1).

The warmed obturating pen tip was used to snip off the extra gutta-percha. Four millimeters below the working length was where the warmed pen tip with gutta-percha was placed. Dentsply Maillefer, Switzerland provided a hand plugger for compacting the warmed gutta-percha. Once more, the obturating pen tip underwent heating and gutta-percha was introduced to approximately half of the root canal's working length. A larger plugger was used for compaction. The canal's opening had been plugged with Cavit (3M ESPE), and the remaining portion was filled with an obturating gun and compressed with a larger plugger. After that, all teeth were kept for a week at 37°C and 100% humidity.

## Measurement of fracture resistance:

Every tooth that had been created was positioned vertically into self-curing acrylic resin blocks that measured 40 mm in diameter and 20 mm in height. Each root's apical 8 mm was left exposed. Before undergoing mechanical testing, the blocks were kept in 100% humidity for a full day after the acrylic resin solidified. Using a Universal Testing Machine, the resistance to fractures was assessed. At the canal orifice, a compressive load was exerted at ninety degree angulation to the tooth's long axis at a speed of 1 mm/min at a 90° angle until fracture happened. Every tooth's fracture force was measured in Newtons (N).

## Statistical analysis:

The data was put in MS excel sheet. The data was presented in the form of Mean±SD. The data was subjected to statistical analysis using SPSS software version 21. Chi square test was used for statistical analysis. P value ≤ 0.05 was considered as statistically significant.

## Results:

There were 5 experimental categories and two controls (positive control and negative control). The fracture resistance (753.24±153.14 N) was maximum in negative control i.e teeth specimens in which neither instrumentation was carried out nor was obturation carried out. The fracture resistance was minimum in positive control (402.30±83.860 N)i.e teeth specimens in which there were instrumentation but no obturation. The fracture resistance in SCO and CLC was 532.15±97.17 N and 548.17±113.34 N respectively. The fracture resistance was greater in CLC as compared to SCO. Similarly, fracture resistance in cold free flow condensation, warm vertical compaction and injection molded thermoplasticized technique was 599.13±117.34 N, 457.35±114.67 N and 407.46±119.42 N. Among experimental categories, maximum fracture resistance was observed in cold free flow condensation technique while minimum fracture resistance was observed in injection molded thermoplasticized technique. The fracture resistance in different obturation techniques was in following order. Negative control> Cold free flow condensation> CLC > SCO > Warm vertical compaction > Injection molded thermoplasticized technique > Positive control. The findings were significant statistically (Table 2).

## Discussion:

This study was conducted to compare the fracture resistance of endodontically obturated teeth in five different obturation techniques. There were 5 experimental categories and two controls (positive control and negative control). The fracture resistance (753.24±153.14 N) was high in negative control i.e teeth specimens in which neither instrumentation was carried out nor was obturation carried out. The fracture resistance was low in positive control (402.30±83.860 N) i.e teeth specimens in which there were instrumentation but no obturation. The fracture resistance in SCO and CLC was 532.15±97.17 N and 548.17±113.34 N respectively. The fracture resistance was greater in CLC as compared to SCO. Similarly, fracture resistance in cold free flow condensation, warm vertical compaction and injection molded thermo-plasticized technique was 599.13±117.34 N, 457.35±114.67 N and 407.46±119.42 N. Among experimental categories, maximum fracture resistance was observed in cold free flow condensation technique while minimum fracture resistance was observed in injection molded thermoplasticized technique. The findings of present study were similar to other studies [13-21].Numerous studies have revealed that removing tooth structure during the instrumentation phase weakens the root and reduces its resistance to fracture [14- 24]. Another study found that fracture resistance was greater in CLC technique as compared to SCO. The findings are similar to findings of our study [15-21]. A number of obturation techniques are available, including single-cone obturation (SCO), thermoplastic gutta-percha, cold lateral compaction (CLC), and vertical condensation [11-19]. The CLC technique is the most commonly used method for obturating root canals [10-15], and it has been shown to produce positive clinical outcomes. However, there are certain drawbacks, such as a high degree of complexity, a chance of void development, and an increased likelihood of vertical fracture of the root since wedging forces are applied using tools like spreaders [11-18]. The CLC approach can also result in less than ideal results if twisted canals are not adequately obturated. Additional area for the placement of supplementary gutta-percha points may be created by properly applying a spreader in the CLC approach [10- 15].

Single cone obturation (SCO)is a variant of the CLC approach [9-14]. This method is well-liked since it is quick and easy to use, and it doesn't require compaction. Since this approach uses increasingly sealer compared to the compaction as well as condensation processes, its results are more dependent on the sealer's characteristics [9-19].In this study, among experimental categories, maximum fracture resistance was observed in cold free flow condensation technique while minimum fracture resistance was observed in injection molded thermoplasticized technique. The fracture resistance in different obturation techniques was in following order.

Negative control > Cold free flow condensation> CLC > SCO > Warm vertical compaction > Injection molded thermoplasticized technique > Positive control.

The findings are similar to observations of some studies [15-24]. A study showed that cold free flow condensation technique had significant greater fracture resistance compared to CLC and SCO. Another study showed that fracture resistance in warm vertical compaction was lesser than CLC and SCO, but greater than injection molded thermoplasticized technique. The findings were similar to findings of present study [21- 25].First decade of twentieth century saw the initial release of GuttaFlow. It is a recently designed flowable, non-heated GP system that combines the features of GP and sealer with the advantages of thermoplasticized GP systems, such as uniform bulk and reduced strains on roots [10-19]. GuttaFlow2, an enhanced GuttaFlow material, preserves the same exceptional material properties. The cold free flow compaction technique serves as its foundation [9-16]. The BeeFill system is a new type of thermoplasticized injection equipment designed to facilitate obturation after canal preparation with the Mtwo rotational system [15-21]. The BeeFill technology is equivalent to a heated vertical compaction system [17-24].Root canal therapy is used to treat the root canal contamination and prevent or stop the periapical spread of infection [11- 18]. Three-dimensional closure of biomechanically prepared root canals is a crucial first step to prevent treatment failure brought on by the reintroduction of bacteria and their by-products into the tooth's canal system and their discharge into the periapical tissue [13-21]. Endodontically treated teeth are weaker and more prone to fracture than healthy teeth because they experience dehydration and dentin erosion during the mechano-chemical preparation process, prolonged pharmaceutical exposure during the disinfection process, and elevated pressure during the obturation process [11-17].Therefore, the goal of root canal obturation is to fortify the root canal and increase resilience to root fractures by adhering to and mechanically linking root canal filling components with root dentin [9-18].Numerous endodontic problems stem from inadequate root canal closure, a study found [14-19]. The combination of gutta-percha and sealer yields the best results for root canal obturation because the former is the most biocompatible substance, doesn't trigger any allergic reactions or negative consequences, and is easy to remove from the root canal system. Its incomplete saturation of root canal space and inability to adhere to dentin, which inhibits it from fortifying roots, are among its disadvantages [24-25].

## Conclusion:

Cold free flow condensation technique using Gutta Flow2 has the potential to be used as obturation technique with minimum fracture resistance. Conventional techniques like CLC and SOC also offered good fracture resistance.

## Figures and Tables

**Table 1:** Details about study categories and distribution of study specimens in these study categories

**Category**	**Obturation technique**	**Corresponding means**	**n**
1	Single cone obturation (SCO)	Single master cone with AH sealer	50
2	Cold lateral compaction (CLC)	Guttapercha with lateral condensation	50
3	Cold free flow condensation	Gutta Flow2	50
4	Warm vertical compaction	Bee Fill (2 in1)	50
5	Injection molded thermoplasticized technique	C-fill system	50
6	Positive control	Although instrumentation was performed, but obturation was not carried out	20
7	Negative control	There was neither instrumentation nor obturation	20

**Table 2:** Fracture resistance (N) values in different study groups

	**Single cone obturation (SCO)**	**Guttapercha with lateral condensation (CLC)**	**Cold free flow condensation**	**Warm vertical compaction**	**Injection molded thermoplasticized technique**	**Positive control**	**Negative control**
Mean value	532.15	548.17	599.13	457.35	407.46	402.3	753.24
SD	97.17	113.34	117.34	114.67	119.42	83.86	153.14
F						17.863	
p						0.001	
